# Numerical simulation data of bubble-structure interactions in near-field underwater explosion

**DOI:** 10.1016/j.dib.2022.108337

**Published:** 2022-06-01

**Authors:** Wentao Ma, Xuning Zhao, Christine Gilbert, Kevin Wang

**Affiliations:** Department of Aerospace and Ocean Engineering, Virginia Polytechnic Institute and State University, Blacksburg, Virginia 24061, USA

**Keywords:** fluid-structure interaction, collapse, bubble dynamics, shock wave, underwater explosion, simulation

## Abstract

The simulation data presented in this paper describes the interaction between a thin-walled aluminum cylinder and a gas bubble in a near-field underwater explosion. The simulation is performed using the AERO-F/S solvers. The finite element AERO-S solver is used to simulate the structural dynamics of the cylinder, including its yielding and collapse. The AERO-F solver is used to simulate the fluid dynamics of the explosion bubble, the surrounding liquid water, and the air inside the cylinder. The two solvers are coupled using an embedded boundary method and the FInite Volume method with Exact two-material Riemann problems (FIVER). The data presented in this paper corresponds to a representative case with initial pressure $p_{0}=12.5\;\text{MPa}$ inside the bubble (cf. [1]). Simulation data include structural stress and deformation, fluid velocity, pressure and bubble dynamics. The input files and the workflow to perform this simulation are also provided. With the information provided in this paper, researchers can repeat this simulation, and use it as a starting point to study related problems involving cavitation bubbles, underwater explosion, and fluid-structure interaction in general.

## Specifications Table

**Table:** 

Subject	Ocean and Maritime Engineering
Specific subject area	fluid-structure interaction, bubble dynamics, underwater explosion, computational mechanics
Type of data	Image Video ASCII files (simulation inputs and outputs)
How the data were acquired	The simulation output data was generated using the AERO-F/S solvers and the Tinkercliffs computer cluster at Virginia Tech.
Data format	Raw
	Visualized
Description of data collection	The simulation was performed using the AERO-F/S solvers. The finite element AERO-S solver was used to simulate the structural dynamics of the cylinder, including its yielding and collapse. The AERO-F solver was used to simulate the fluid dynamics of the explosion bubble, the surrounding liquid water, and the air inside the cylinder. The images and videos were generated using ParaView 5.8.1.
Data source location	• Institution: Virginia Polytechnic Institute and State University
	• City/Town/Region: Blacksburg, VA
	• Country: USA
Data accessibility	Repository name: Mendeley Data
	Data identification number: 10.17632/8jbfz89rbp.1
	Direct URL to data: https://data.mendeley.com/datasets/8jbfz89rbp/1
Related research article	[1] W. Ma, X. Zhao, C. Gilbert, K. Wang, Computational analysis of bubble-structure interactions in near-field underwater explosion, International Journal of Solids and Structures 42 (2022) 111527.
	doi: https://doi.org/10.1016/j.ijsolstr.2022.111527

## Value of the Data

• The simulation data describe a complex dynamic interaction between a thin-walled aluminum cylinder and a high-pressure gas bubble that represents the bubble generated by an underwater explosion. The simulation accounts for large structural deformation, yielding, plastic deformation, and self-contact. It also accounts for the compressibility of the gas bubble and the surrounding liquid water.
• The simulation predicts a counter-intuitive mode of collaspe, in which the closest point on the cylinder to the explosive charge moves towards the charge.
• The data presented in this manuscript allows researchers to repeat the simulation, which is a representative case in the co-submitted paper [1].
• The simulation input files can be modified to study related problems involving cavitation bubbles, underwater explosion, and fluid-structure interaction.

## 1 Data Description

This paper presents a set of data associated with the numerical simulation of the interactions between a high-pressure gas bubble and a thin-walled aluminum cylinder, in the context of near-field underwater explosion. The fluid dynamics is simulated using finite volume method implemented in the AERO-F solver [2]. The structural dynamics is simulated using finite element method implemented in the AERO-S solver [3]. The two solvers are coupled using an embedded boundary method and the FInite Volume method with Exact two-material Riemann problems (FIVER) [4], [5], [6].

**Fig. 1:**
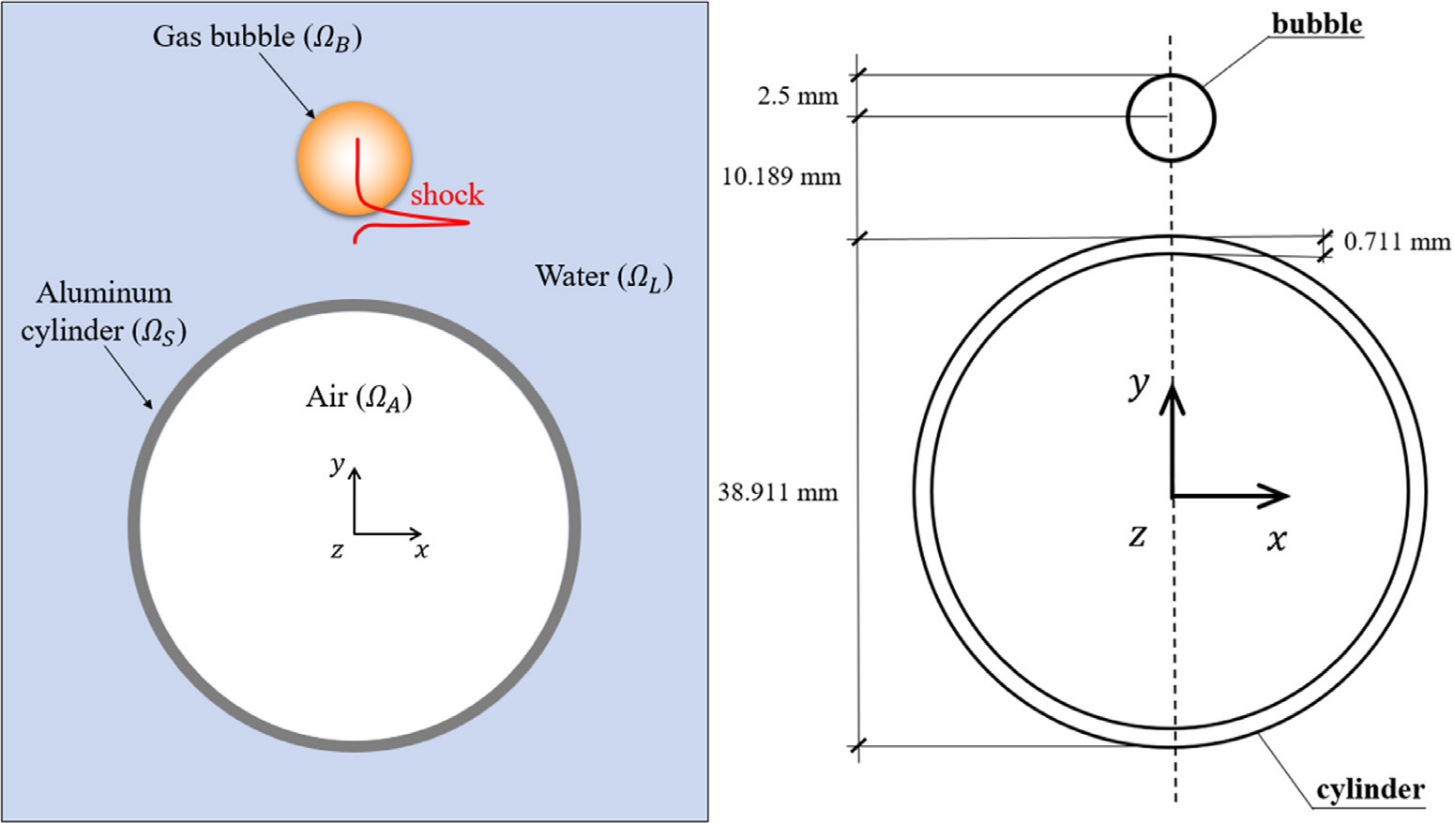
Setup of the numerical experiment [1].

Fig. 1 (a) illustrates the problem investigated in this work. An air-filled thin-walled aluminum cylinder is submerged in water. A gas bubble with high initial pressure is located near the cylinder. Because of the strong discontinuity in pressure across the bubble surface, the bubble expands rapidly at the beginning of the numerical experiment and emits a shock wave that impacts the cylinder. Afterwards, the bubble continues to expand and contract, and interacts with the surrounding water and the cylinder. The detailed setup of the numerical experiment is shown in Fig. 1(b).

Table 2 presents the files that have been uploaded to the online repository, including the input files that are required to launch the simulation and selected simulation outputs. The file paths are relative to the main directory. Specifically, the input files are placed inside the *Simulation* folder. A sequence of 626 image files are placed in the *Images* folder, which shows the evolution of the structural deformation, bubble dynamics, and the fluid velocity and pressure fields. An animation created using these images is located in the *Video* folder. The screen outputs generated by the simulation are recorded in a file, and placed inside the *Simulation* folder.

**Table 2:** Simulation input and output files.

File path	File description
Simulation/mesh.include	The structural mesh
Simulation/fem.in	Input parameters for the structural dynamics solver (AERO-S)
Simulation/fluid2d.top	The fluid mesh
Simulation/fluid2d.top.dec.639	A partition of the fluid mesh for parallel computation (639 parts)
Simulation/input.st	Input parameters for the fluid dynamics solver (AERO-F)
Simulation/tinkercliffs_sbatch.sh	The bash script for submitting the simulation on Tinkercliffs
Simulation/slurm-133660.out	The screen outputs generated by the simulation
Images.zip	A sequence of images generated using the simulation data
Video/12.5MPa.avi	An animation of the simulation data

## 2 Experimental Design, Materials and Methods

### 2.1 Geometric and material properties

Table 3 presents the material and geometric properties of the aluminum cylinder, which are the same as the ones used in the validation study in [7]. The physical properties of the gas bubble are listed in Table 4. The properties of the ambient water and the air inside the cylinder are listed in Table 5.

**Table 3:** Material and geometric properties of the cylinder (Aluminum 6061-T6) [1].

Young’s modulus	Poisson’s ratio	Density	Yield stress	Tangent modulus	Outer diameter	Thickness
69.6 GPa	0.33	2779 kg/${\text{m}}^{3}$	292 MPa	674 MPa	38.911 mm	0.711 mm

**Table 4:** Bubble properties [1].

Stand-off distance	Initial radius	Initial density	Initial pressure	Heat capacity ratio
10.189 mm	2.5 mm	50.0 kg/m${}^{3}$	12.5 MPa	1.4

**Table 5:** Properties of the ambient water and the air inside the cylinder [1].

Water pressure	Water density	Air pressure	Air density	Air heat capacity ratio
1.0 MPa	1000.39 kg/m${}^{3}$	0.1 MPa	1.225 kg/m${}^{3}$	1.4

### 2.2 Solvers and external libraries

The simulation was performed using *changeset* 2101:7f9049f89e19 in the AERO-F repository [8] and *changeset* 3152:f484d5c512c8 in the AERO-S repository [9]. The versions of external libraries used by the AERO-F/S solvers are listed in Table 6.

**Table 6:** External libraries used by AERO-F and AERO-S.

Name	Version
Boost	1.71.0
Intel MPI	2018.5.288
Eigen	3.3
METIS	5.1.0
MUMPS	5.2.1

### 2.3 Simulation process

The fluid domain is divided into 639 subdomains, with each one assigned to one CPU core. The simulation parameters are specified in *input.st* and *fem.in*. Detailed information about each parameter can be found in the manuals of AERO-F and AERO-S [2], [3].

Before launching the simulation, the fluid mesh and partition files were converted into 639 binary files, each one containing the mesh information of one subdomain. This was done using the SOWER program [10]. The simulation was launched on the Tinkercliffs computer cluster using the sbatch script *tinkercliffs_sbatch.sh*.

**Fig. 2:**
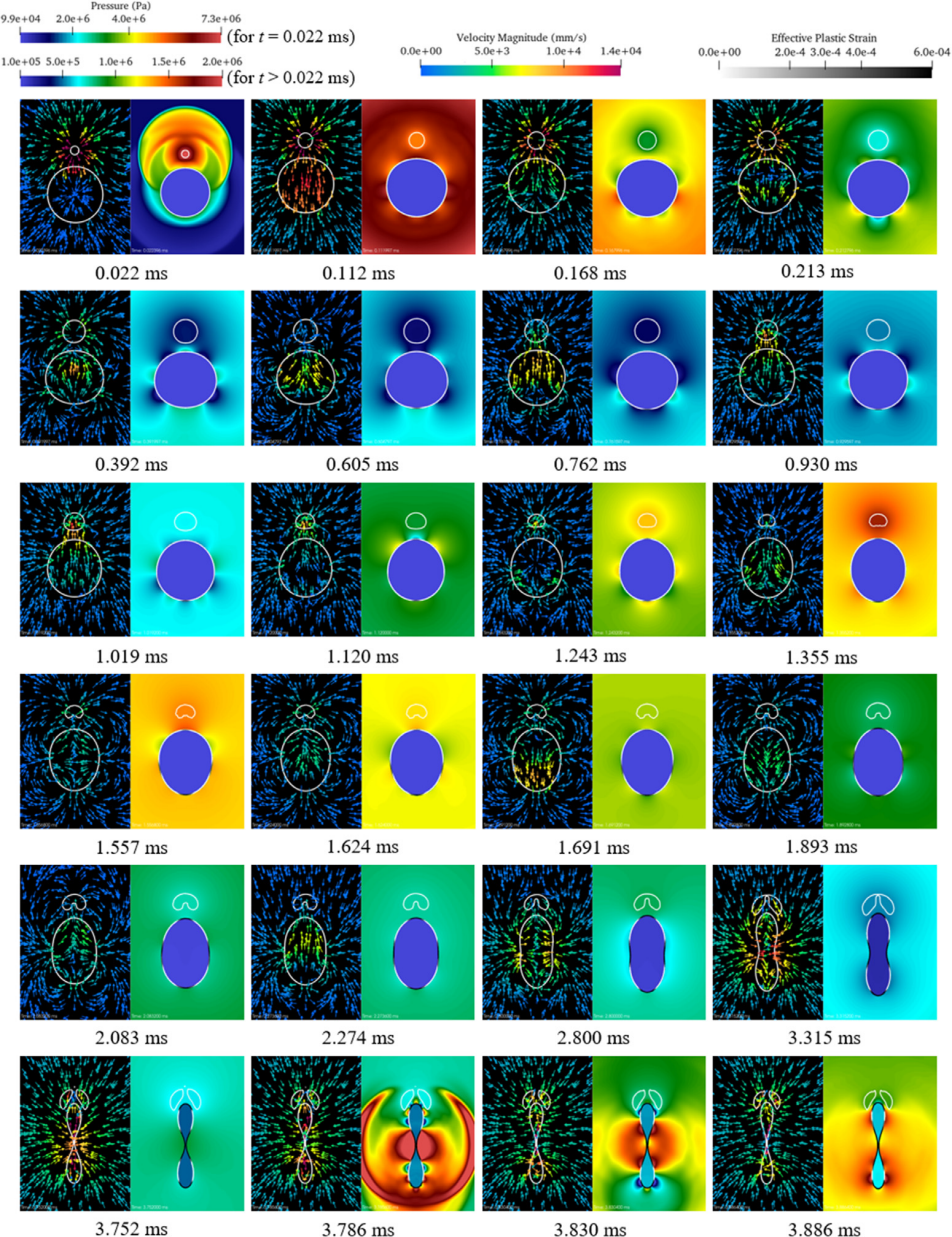
Solution snapshots of the fluid velocity and pressure fileds and the cylinder’s deformation.

The simulation was performed using 640 CPU cores. The time step size was 7ns. After 857,143 time steps (t=0.006s), the simulation was terminated. The total wall-clock time was approximately 23.2 hours.

### 2.4 Simulation data

Outputs of the simulation include, but are not limited to, the fluid pressure, velocity, and level-set (for liquid-gas interface tracking) fields and the structural displacement, velocity, stress, and strain fields. Fig. 2 presents a sequence of images showing the simulated fluid-structure interaction process. In Fig. 2, each sub-figure is taken at a time instant labeled at the bottom. Within each sub-figure, the fluid velocity and pressure fields are shown in the left and right halves, respectively. In addition, the cylinder’s effective plastic strain is shown in the right half of each sub-figure.

Additional data in the form of images and animation can be found in the uploaded folder. All the images were generated using ParaView, version 5.8.1.

## Ethics Statements

This work does not involve human subjects, animal experiments, or data collected from social media platforms.

## CRediT authorship contribution statement

**Wentao Ma:** Conceptualization, Methodology, Investigation, Visualization, Software, Writing – original draft, Writing – review & editing. **Xuning Zhao:** Investigation, Visualization, Writing – review & editing. **Christine Gilbert:** Resources, Writing – review & editing. **Kevin Wang:** Conceptualization, Methodology, Software, Writing – original draft, Writing – review & editing, Project administration.

## Declaration of Competing Interest

The authors declare that they have no known competing financial interests or personal relationships that could have appeared to influence the work reported in this paper.

## Data Availability

Numerical simulation data of bubble-structure interactions in near-field underwater explosion (Mendeley Data).

## References

[1] Ma W., Zhao X., Gilbert C., Wang K. (2022). Computational analysis of bubble - structure interactions in near-field underwater explosion. International Journal of Solids and Structures.

[2] Farhat Research Group (2017).

[3] Farhat Research Group (2017).

[4] Wang K., Rallu A., Gerbeau J.-F., Farhat C. (2011). Algorithms for interface treatment and load computation in embedded boundary methods for fluid and fluid-structure interaction problems. International Journal for Numerical Methods in Fluids.

[5] Farhat C., Gerbeau J.-F., Rallu A. (2012). Fiver: A finite volume method based on exact two-phase riemann problems and sparse grids for multi-material flows with large density jumps. Journal of Computational Physics.

[6] Main A., Zeng X., Avery P., Farhat C. (2017). An enhanced FIVER method for multi-material flow problems with second-order convergence rate. Journal of Computational Physics.

[7] Farhat C., Wang K., Main A., Kyriakides S., Lee L.-H., Ravi-Chandar K., Belytschko T. (2013). Dynamic implosion of underwater cylindrical shells: experiments and computations. International Journal of Solids and Structures.

[8] Farhat Research Group, AERO-F Repository, Stanford University, Accessed Februray 4, 2022, https://bitbucket.org/frg/aero-f/src/master/.

[9] Farhat Research Group, AERO-S Repository, Stanford University, Accessed Februray 4, 2022, https://bitbucket.org/frg/aero-s/src/master/.

[10] Farhat Research Group, SOWER Repository, Stanford University, Accessed Februray 4, 2022, https://bitbucket.org/frg/sower/src/master/.

